# The Occurrence of *Chlamydia felis* in Cats and Dogs in Hungary

**DOI:** 10.3390/pathogens13090771

**Published:** 2024-09-06

**Authors:** Áron Balázs Ulbert, Hajnalka Juhász, Zsanett Karácsony, Katalin Bencze, Zoltán Deim, Katalin Burián, Gabriella Terhes

**Affiliations:** 1 Department of Medical Microbiology, Albert Szent-Györgyi Medical School, University of Szeged, 6725 Szeged, Hungary; ulbertaron@gmail.com (Á.B.U.); hajnus2007@gmail.com (H.J.); karacsony.zsanett@med.u-szeged.hu (Z.K.);; 2 Oxygen Animal and Environment Foundation, 6721 Szeged, Hungary; oxigen.asegitseg@gmail.com; 3 Dr. Zoltán Deim Veterinary Clinic, 6726 Szeged, Hungary; info@dmac.hu

**Keywords:** *Chlamydia felis*, chlamydiosis, animal health, one health, zoonotic diseases

## Abstract

The World Health Organization (WHO) estimates that many human infections are zoonoses, creating a worldwide public health challenge. Among *Chlamydia* species, *Chlamydia felis* is the leading cause of conjunctivitis in cats and is a prominent zoonotic species. This study aimed to determine the occurrence and risk of chlamydiosis in cats and dogs in Szeged, Hungary, and surrounding areas. The total nucleic acids from conjunctival swab samples of symptomatic and asymptomatic animals were extracted using an automated nucleic acid extraction system. After that, DNA was amplified by pan-chlamydia PCR. Bacterial and fungal cultures were also performed to detect other microorganisms. Of the 93 animals, 32 (34.4%) were positive for pan-chlamydia PCR. The positivity rates were 33.3% (26/78) in cats and 40.0% (6/15) in dogs. Furthermore, the positivity rates were 37.2% (16/43) in the cat shelter, 42.4% (14/33) in the veterinary clinic, and 11.7% (2/17) in household pets. In total, 103 species were identified through culture-based examinations, including 97 (94.2%) bacterial and 6 fungal (5.8%) species. From both human and animal health perspectives, it is essential to have a detailed understanding of the circumstances of chlamydiosis, given the global impact of zoonotic diseases.

## 1. Introduction

The One Health concept has become a globally accepted approach to detecting and managing zoonoses in the past decade. This concept emphasizes the importance of multisectoral collaboration and developing action plans for preventing zoonotic infections. This is particularly crucial given that, according to estimates by the World Health Organization (WHO), many human infections are of zoonotic origin, posing substantial public health challenges worldwide. Beyond direct contact with animals, zoonoses can also present severe risks to the international trade and production of animal-derived products [1,2,3].

The *Chlamydia* genus comprises numerous obligate intracellular zoonotic pathogens that infect vertebrate animals, particularly mammals, birds, and reptiles [4,5,6,7,8]. In addition to infections in wild animals, species such as *C. suis*, *C. abortus*, *C. pecorum*, and *C. psittaci* also infect various livestock. *C. felis* and *C. caviae* typically infect household pets, such as cats and guinea pigs, and these infected pets can potentially be a source of infection for their owners [7,8,9]. Although *Chlamydia pneumoniae* is primarily a common respiratory pathogen in humans, it can also infect many animals, including horses, cattle, cats, dogs, and various reptiles and amphibians [8]. In animals, chlamydiosis can cause atypical pneumonia, enteritis, conjunctivitis, and endocarditis, with *C. abortus* and *C. psittaci* particularly causing intrauterine fetal death in mammals and birds [4,5].

In addition to the species mentioned above, the zoonotic potential of *C. felis* needs to be considered. This pathogen most commonly infects cats’ upper respiratory tract and eyes, causing respiratory symptoms and conjunctivitis. Infected cats spread the pathogen through secretions from these locations [4].

According to the literature, dogs can also become infected, presenting clinical signs similar to those observed in cats [4]. However, reports of canine chlamydiosis are not common. Some studies have identified *C. felis* infections in asymptomatic dogs by real-time PCR assays. Unlike cats, dogs infected with *C. felis* rarely develop conjunctivitis [4,10,11]. In seroprevalence studies conducted in China, antibodies to *C. felis* were found in 32 (12.1%) of 264 pet dogs, which was higher than the previously reported result (2.87%) in Dongguan, southern China [4,12].

Both dogs and domestic cats are increasingly common household pets; however, it is crucial to be aware that these animals, primarily cats, may be sources of chlamydial infections [4,13,14]. Zoonotic transmissions are rarely reported, except for some cases, demonstrating its potential risk for zoonotic infections in humans with close contact with diseased animals in particular [9]. A novel study summarizing three cases reported that, in three patients with clinical signs of conjunctivitis, living in close contact with their cats, *C. felis* was detected. The findings demonstrate that zoonotic infections in atypical conjunctivitis require specific PCR testing for diagnosis and show that, instead of azithromycin, doxycycline is more effective for treatment, as in the case of cats [15].

Currently, no data on the occurrence of various zoonotic *Chlamydia* species, including *C. felis*, are available in Hungary, and no comprehensive study has been conducted on the occurrence of human diseases. Based on this, our study aimed to determine the regional occurrence of *C. felis* infections in symptomatic and asymptomatic cats and dogs using molecular methods supplemented with bacteriological and fungal cultures.

## 2. Materials and Methods

### 2.1. Sample Collection

Between July 2022 and October 2023, conjunctival swab samples were collected from symptomatic and asymptomatic cats and dogs. Samples were collected in Szeged and its surrounding urban and peri-urban areas (within a 5 km radius of Szeged). The town is in southern Hungary, close to western Romania’s and northern Serbia’s borders. Sample collection was performed considering three types of pets: pets in a veterinary clinic, those in a cat shelter, and household pets. In the veterinary clinic, animals, including cats and dogs with conjunctivitis, were sampled; in the cat shelter, symptomatic and asymptomatic cats were involved in the study (Oxygen Animal and Environmental Protection Foundation); and in the last category (household pets), swabs were taken from symptomatic and asymptomatic cats and symptomatic dogs, whose owners volunteered to participate in this study. In the case of symptomatic animals, clinical signs could be observed, including excessive tearing from one or both eyes, mucopurulent discharge, and inflamed conjunctival membranes. During sample collection, conjunctival swab samples were taken from the eyes of both cats and dogs by gently pulling down the eyelid, with particular attention to minimizing the duration and invasiveness of the procedure to ensure the animals’ comfort. Two swab devices were used: Transwab (MWE.CO., Corsham, UK) for culture-based tests and Citoswab (Citotest Labware Manufacturing Co., Ltd., Nanjing, China) for molecular tests. A total of 101 samples were collected from 93 animals.

### 2.2. Molecular Detection of Chlamydia Infections

**Figure 1:**
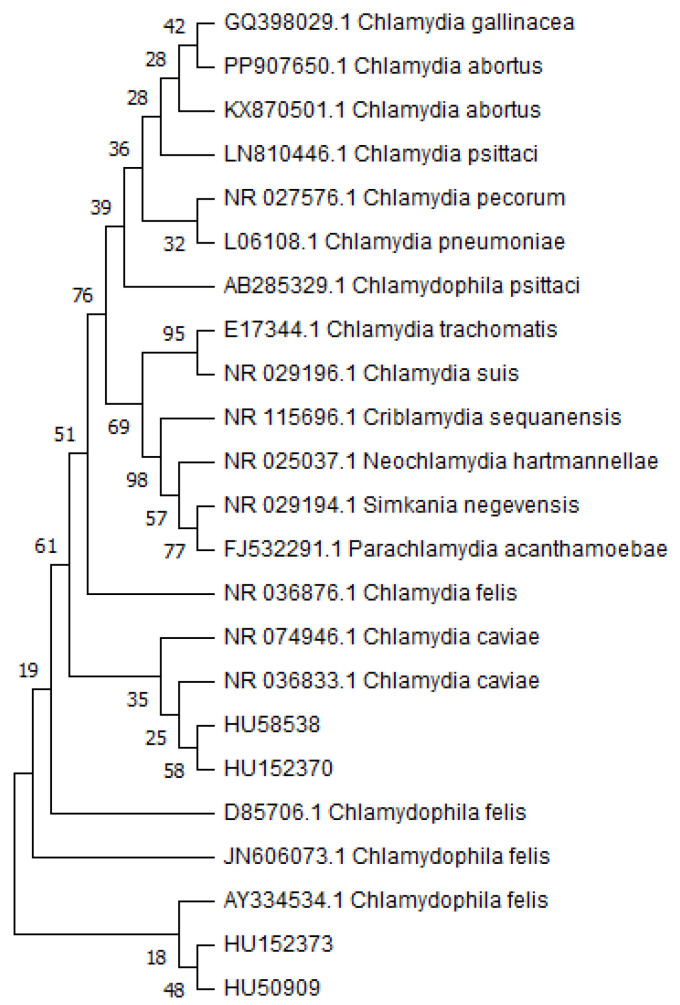
Phylogenetic tree based on the sequence analysis of pan-chlamydial PCR product. HU58538, HU152370, HU152373, and HU50909 are indicated as Hungarian data.

Nucleic acid extraction from conjunctival swab samples was performed using the MT-Prep^TM^ Viral/Pathogen Nucleic Acid Extraction Kit B (AusDiagnostics, Mascot, Australia) according to the manufacturer’s instructions on the MT-Prep^TM^ 24 instrument (AusDiagnostics, Mascot, Australia). Bacterial DNA was amplified using real-time PCR with *Chlamydia* genus-specific primers. The PCR reaction mixture for each sample was set to a final volume of 20 μL, comprising 10 μL 2× Sybr Green Master Mix (Thermo Fisher Scientific, Waltham, MA, USA); 0.2 μL 25 pmol Ch primer F (5′-CCGCCAACACTGGGACT-3′) [16]; 0.2 μL 25 pmol Ch primer R (5′-GGAGTTAGCCGGTGCTTCTTTAC-3′) [16]; 0.4 μL 25 mM MgCl_2_ (Thermo Fisher Scientific, Waltham, MA, USA); 4.2 μL nuclease-free water (Thermo Fisher Scientific, Waltham, MA, USA); and 5 μL nucleic acid template. The PCR conditions were as follows: initial denaturation (10 min, 95 °C) followed by 45 cycles of denaturation (15 s, 95 °C) and annealing (1 min, 58 °C). The PCR product was a fragment of about 207 to 215 bp, and all real-time positive PCR products were checked by agarose gel electrophoreses. The Gentier96E real-time PCR instrument (Xian Tianlong Science and Technology Co., Ltd., Xi’an, China) was used for real-time PCR. Upon obtaining a positive result (Ct (cycle threshold) value less than 30), a second reaction (total volume of 100 µL) was set up for PCR product sequencing, with the product verified on 1.5% agarose gel using ECO Safe Nucleic Acid Staining Solution (Pacific Image Electronics, Taiwan, China). According to the manufacturer’s instructions, the PCR product was purified from agarose gel using the GeneJET Gel Extraction Kit (Thermo Fisher Scientific, Waltham, MA, USA). PCR products were sequenced using the GenomeLab DTCS—Quick Start Kit (Beckman Coulter, Indianapolis, IN, USA), and sequences were compared with those available in GenBank using NCBI BLAST (Nucleotide Blast; default settings, standard database, optimized for highly similar sequences). The phylogenetic tree was constructed with MEGA11 using the maximum likelihood method (bootstrap values 1000) (Figure 1). Because of the high sequence homologies in the sequenced PCR products, differentiation between *C. felis* and *C. caviae* was not definite (BLAST search gave the same identification score for both species); thus, species-specific PCR was set up, and all pan-chlamydia-positive samples were checked using this PCR. We used MOMP gene real-time PCR in this case, according to Helps et al. (2001) [17].

### 2.3. Culture-Based Examinations

The detection of bacterial and fungal species other than *C. felis* from conjunctival swab samples was also part of the study. Culture-based examinations were conducted on chocolate agar (PolyViteX, bioMérieux SA, Marcy-l’Étoile, France), Schaedler agar (bioMérieux SA, Marcy-l’Étoile, France), Columbia agar (bioMérieux SA, France), and Sabouraud Chloramphenicol agar (Bio-Rad, Mitry-Mory, France). Following inoculation, Sabouraud plates were incubated under a normal atmosphere for 24 h at 36 ± 1 °C, while chocolate and Columbia agars were incubated at 36 ± 1 °C in a 5% CO_2_ incubator for the same duration. Shaedler agar was incubated anaerobically (Whitley A45 workstation, Don Whitley Scientific, Bingley, UK) at 36 ± 1 °C for 48 h. Cultured microorganisms were identified using the MALDI Biotyper^®^ Sirius system (Bruker, Billerica, MA, USA).

### 2.4. Veterinary Treatment Details

In the case of symptomatic animals with positive chlamydia PCR results, the following treatment was applied: The veterinarian administered oral doxycycline hyclate therapy for 7, 10, 14, and 21 days. The dosage was 100 mg for animals up to 15 kg and 2 × 100 mg for animals over 15 kg. Rifampicin eye drops were also applied for the same duration. Treatment continued until complete recovery, often supported by negative PCR results upon the veterinarian’s request.

## 3. Results

### 3.1. Summary of Sample Collection

Between July 2022 and October 2023, we examined 101 conjunctival swab samples from 93 animals. Table 1 presents detailed results of the sample collection.

We collected 43 samples from the cat shelter, 42 samples from the veterinary clinic (from 33 animals), and 17 samples from animals in our circle of acquaintances. Given the focus on chlamydial infection primarily affecting cats, cat samples predominated. In total, 78 (83.8%) cats and 15 (16.1%) dogs were included in this study. Of these, 56 (60.2%) animals showed clinical signs, while 37 (39.8%) were asymptomatic. All the dogs in this study were symptomatic. Samples from the veterinary clinic were all obtained from symptomatic animals, whereas those from the cat shelter and household pets included samples from symptomatic and asymptomatic individuals. In this study, clinical signs always manifested as conjunctivitis. Multiple samplings were conducted to monitor treatment success, which occurred in six (6.5%) animals (14 samples), comprising five dogs and one cat.

### 3.2. Detection of Chlamydiosis

Of 101 conjunctival swab samples, 33 (32.7%) were positive by pan-chlamydia PCR. These samples originated from 32 animals, with a second sample from 1 cat yielding a positive PCR result due to veterinary follow-up. Thus, out of 93 animals, 32 (34.4%) tested positive by PCR. Detailed data of individuals with positive pan-chlamydia PCR results are presented in Table 1. 

This group includes both chlamydia-infected animals and asymptomatic carriers. From the cat shelter, 16 (17.2%); from the veterinary clinic, 14 (15.0%); and from the household pet category, 2 (2.2%) animals tested positive by PCR. Among them, 19 (20.4%) were symptomatic, and 13 (14.0%) were asymptomatic. Positivity rates were 33.9% (19/56) in symptomatic cases, and 35.1% (13/37) in asymptomatic cases. Positivity rates were 37.2% (16/43) in the cat shelter; 4 of 8 symptomatic and 12 of 35 asymptomatic cats proved positive by pan-chlamydial PCR. Notably, 42.4% of animals in the veterinary clinic (cats 14/33, and dogs 6/13) and 11.7% of animals in the household pet category gave pan-chlamydia PCR positive results. Pan-chlamydial PCR positivity rates were 33.3% (26/78) in cats (symptomatic cats 13/41, asymptomatic cats 13/37), and 40.0% (6/15) in dogs. Based on our findings, the proportion of asymptomatic individuals with positive pan-chlamydial results was higher at the cat shelter, and the rate of symptomatic individuals was higher in the veterinary clinic and the household pet category. This is attributed to the fact that animals at the clinic arrived with clinical signs to initiate veterinary care. In the household pet category, samples from owners were usually sent for testing when clinical signs were observed in the animals. Often, these samples were from untreated animals that had not received veterinary care.

Because of the high degree of homology in the sequence of *Chlamydia* spp. using 16S rDNA sequencing, besides sequencing, we set up *C. felis*-specific PCR in all pan-chlamydia-positive samples. Notably, 13 out of 33 pan-chlamydia-positive specimens gave positive results using MOMP gene real-time PCR. Eleven samples were collected from cats (nine from the cat shelter, one from the household pet category, and one from the veterinary clinic) and two samples originated from dogs (both of them were treated in the veterinary clinic). In the case of 4 out of 13 samples, the sequence of pan-chlamydia PCR products was determined.

The preparation and sequencing of PCR-positive samples for genotyping were carried out. Samples with Ct values above 30 were excluded due to insufficient PCR product quantity, rendering them undetectable during preparation for sequencing. Therefore, sequencing was conducted only on samples with Ct values below 30 and if the agarose gel electrophoresis gave adequate results after PCR product purifications. Out of the 33 samples, 4 (12.1%) met this criterion. The sequencing of the PCR product showed a close genetic relationship between *C. felis* and *C. caviae* (Figure 1); thus, these results had to be confirmed by *C. felis*-specific PCR, which gave positive results in all four cases. In the case of sequencing, two samples were collected from symptomatic cats and two from symptomatic dogs; pan-chlamydial PCR gave results with Tm values ranging from 81.6 to 82.1 (Figure S1).

### 3.3. Results of Culture-Based Examinations

In addition to detecting chlamydiosis, we searched for other microorganisms from conjunctival swab samples. Therefore, concurrent culture-based examinations were conducted. A detailed summary of identified bacteria and fungi can be found in Supplementary File S1. Of the 153 microorganisms identified from the 93 samples, colonies did not grow on any medium in 27 cases; thus, these cultures were considered negative. Culture-negative samples were collected from 10 symptomatic cats, 11 asymptomatic cats, and 6 symptomatic dogs; pan-chlamydia PCR gave positive results in 10 of 27 cases.

Of the 153 microorganisms, 146 (95.4%) were bacteria and 7 (4.6%) were fungi. A total of 103 different species were identified, comprising 97 (94.2%) bacterial species belonging to 42 different genera and 6 (5.8%) fungal species belonging to 6 fungal genera. 

*Pseudomonas* was the most common genus, with 17 (11.1%) bacteria identified, representing 15 species. Within this genus, *Pseudomonas koreensis* was the most frequent species (n = 5, 29.4%). *Staphylococcus*, *Acinetobacter*, *Microbacterium*, *Enterococcus*, and *Bacillus* genera were more frequent than the average (2.1%). *Staphylococcus felis* (n = 7, 4.6%) was the most common species identified.

For fungi, except for the *Malassezia* genus (n = 2, 1.3%), one species per genus was identified. Among the fungal isolates, only *Aspergillus flavus* was isolated from an asymptomatic pet.

Determining whether the isolated strain is pathogenic or colonizes the ocular surface is often difficult. However, it was clear that based on our findings in the case of colonization, the number of isolated strains was lower (48 strains) than in the case of cats with clinical signs referring to ocular infections (105 strains were isolated). The most frequent bacterial genus in symptomatic pets was *Pseudomonas*, followed by *Staphylococcus*. *Enterococcus* (8/9 strains) and *Microbacterium* (8/10 strains) genera were also frequently associated with ocular inflammation. *Klebsiella* sp., *Pantoea* sp., and *Bacillus* sp. were cultured only from symptomatic animals. In contrast, in the case of *Acinetobacter* sp., seven isolates were cultured from symptomatic pets, and eight isolates originated from asymptomatic pets.

### 3.4. Veterinary Treatment of Chlamydia sp. Infection

The veterinarian initiated treatment upon confirming *Chlamydia* spp. infection suspicion. In some cases, repeated PCR examinations were requested to monitor therapy. This was performed for four dogs and two cats. Typically, one repeat examination sufficed for most animals, although three repeat examinations were required for one cat to obtain a negative PCR result and the resolution of clinical signs. The veterinarian confirmed full recovery occurred by the 14th day of treatment, evidenced by symptom resolution and negative results from pan-chlamydia PCR examinations.

## 4. Discussion

This study aimed to determine the regional prevalence and evaluate the risk of chlamydial zoonoses in cats and dogs. In this study, 32 (34.4%) animals tested positive for pan-chlamydia PCR. Out of these, 19 (20.4%) animals were symptomatic, and 13 (14.0%) were asymptomatic, indicating a significant proportion of symptomatic animals among those with positive PCR results. Following *C. felis*-specific PCR and sequencing, it was confirmed in thirteen cases (four pan-chlamydial PCR products were sequenced) that the pathogen was *C. felis.* Sequencing results showed a close genetic relationship between *C. caviae* and *C. felis* (Figure 1). According to the literature, *C. caviae* is one of guinea pigs’ most common infectious agents causing conjunctivitis. The pathogen has also been detected in cats and dogs, but it has not been associated with the development of clinical signs in these animals. In our study, all four sequenced PCR products were from individuals exhibiting clinical signs of conjunctivitis. For all these reasons, and due to the results of specific PCR, the pathogenic role of *C. caviae* can be excluded in these cases [18,19]. In the case of these four samples, animals had severe clinical signs, which may correlate with low Ct values of pan-chlamydia PCR. However, more samples are necessary to make conclusions because, in one case (ID 50911), severe inflammation led to total blindness, and later, the infected eye was removed; in this case, pan-chlamydia Ct was 38.10, the sample was also positive for *S. felis* and *Pantoea* sp. The severity of clinical signs may be influenced by bacteria or fungal coinfection, the general condition of animals, and environmental factors. The influence of the environment where the animals reside on the severity of *C. felis* infection was also confirmed by Gonsales et al. [20]. Of the 56 symptomatic animals, 19 (33.9%) tested positive for pan-chlamydia PCR, suggesting that in the remaining 37 (66.1%) animals, *Chlamydia* sp. was likely not responsible for the clinical signs. This indicates the possibility of other infections, such as feline herpesvirus and calicivirus in cats or canine herpesvirus in dogs, or bacterial and fungal infections [21,22]. Further investigations are needed to determine the causes of clinical signs in these cases.

A previous study found that the prevalence of *C. felis* infection may be significantly higher in cats younger than one year compared to those older than one year [23]. *C. felis* is the main *Chlamydia* species associated with cats; other chlamydial species, particularly *C. abortus*, *C. pneumoniae*, *C. psittaci*, *C. suis*, and *C. caviae*, have occasionally been reported [7,10,24,25,26]. Moreover, symptoms such as conjunctivitis in cats and dogs caused by other *Chlamydia* species, more precisely *C. psittaci* and *C. pneumoniae*, have been reported [25,26]. According to several recent studies from different countries (using PCR, DNA microarray, isolation, or immunofluorescence assays), the chlamydial prevalence in pet cats ranges from 0% to 10% in asymptomatic animals and 5.6% to 30.9% in cats with conjunctivitis. In stray cat populations, the prevalence is typically higher, with overall positivity rates of 24.4% to 35.7%, but it can reach up to 65.8% in subgroups with conjunctivitis [7]. In the most recent studies in China, the positivity rate was 11.76% in symptomatic stray cats (higher in Jiading District: 23.53%) and 11.62% in symptomatic domestic cats [27,28].

Among the 37 asymptomatic animals, 13 (35.1%) tested positive for PCR. These animals were considered asymptomatic carriers. Such carriers were predominantly found among cats in the cat shelter (34.3%). The high proportion of asymptomatic carriers is noteworthy because they can easily maintain the infection within the population, as they do not receive treatment due to the absence of clinical signs. Therefore, newly admitted, injured, immunosuppressed, or even healthy animals at the cat shelter are at higher risk of infection. Additionally, these carriers pose a greater risk to humans, as they can transmit the infection unnoticed. While these scenarios can also occur in symptomatic animals, they are more likely to be treated. A crucial aspect of this study is emphasizing that the possibility of chlamydiosis, including *C. felis* infections, should be considered more frequently in professional settings, alongside the more commonly occurring herpesviruses that cause conjunctivitis [21,22,29,30]. This awareness can lead to appropriate treatment, reducing the disease and the risk of further infections. Thus, despite all samples from the veterinary clinic originating from symptomatic animals, the proportion of PCR-positive cases did not exceed that of the shelter animals, which included both symptomatic and asymptomatic individuals. This result also indicates a high occurrence of asymptomatic carriers within the shelter population. Bressan et al. (2021) published an article in which conjunctival and rectal samples of Swiss stray and pet cats were analyzed, and they compared their results with recent international studies. Based on their comparison, our results fall within the range of chlamydiosis detected, namely symptomatic pet cats (5.6% to 30.9%) and stray cats (24.4% to 35.7%), and it can reach up to 65.8% in subgroups with conjunctivitis [7]. Bressan et al. found that, in Swiss stray cats, 19.1% of the stray cats and 11.6% of pet cats were positive for *Chlamydiaceae*. A higher rate was detected in cats with conjunctivitis compared to healthy cats (6.9%) [7]. As the conditions of shelter cats and stray cats are essentially similar, the shelter group in our study (37.2%) was compared to the stray cats. Of all the relevant subgroups, the highest positivity rate, similar to that recorded by Bressan et al., was observed in symptomatic shelter cats, at 50.0% [7]. In Central Europe, data on the occurrence of chlamydial zoonoses are limited. In Romania, a study similar to ours was conducted. It focused exclusively on stray cats. Of 95 cats, 62 (65.3%) tested positive for *Chlamydia* spp. by PCR. Among these PCR-positive cats, 45 (72.6%) were asymptomatic, and 17 (27.4%) showed clinical signs of conjunctivitis [31]. This study reported a much higher (approximately double) proportion of PCR-positive cases than our findings. In the Romanian study, PCR-positive results were most common in asymptomatic individuals, whereas in our study, the proportion of asymptomatic PCR-positive cases was lower. The differences could be attributed to the fact that the Romanian study exclusively collected samples from stray cats, making the sample sources not entirely comparable. The closest similarity was found in the shelter cat population, consisting of animals captured under conditions similar to stray cats. If we only consider this group for PCR-positive cases, the proportion of asymptomatic individuals was 75% (12/16), three times that of symptomatic instances, at 25% (4/16). This closely resembles the results of the above-mentioned Romanian study. Another study conducted in Slovakia found that *C. felis* PCR-positive results were over seven times higher in cats with clinical signs compared to asymptomatic cats. This study included 140 cats categorized into four main groups: strictly indoor cats, outdoor domestic cats, stray cats captured from the street, and shelter cats. In our study, there was not such a large difference between the positivity rates of asymptomatic and symptomatic individuals and their subgroups. However, similar to our results, the highest positivity rates were observed in the stray cat (35.7%) and shelter cat (31%) populations, when compared to the other categories [32]. These findings suggest that different animal populations from various environments show varying positivity rates. However, it is consistently clear that the highest PCR positivity rates were observed in shelter cats and stray cats.

It is essential to gain more information regarding the composition of the resident and transient normal flora of the ocular surface and possible opportunistic pathogens to determine the causative role of the isolated species, outline the treatment, and decrease unnecessary antibiotic use because, in many cases, the members of the normal flora are the same as causative agents of eye infections [33,34,35]. Our results confirmed earlier findings that pets’ most important pathogens of conjunctivitis are *Pseudomonas*, *Staphylococcus*, *Enterococcus*, *Klebsiella*, *Pantoea*, and *Bacillus* [33,34,35,36]. A high colonization rate (27 (26.7%)/101 samples) could be observed in our study, which is also similar to earlier findings [33]. Among the bacteria, members of the *Bacillus*, *Enterobacteriales*, *Pseudomonas*, *Clostridium*, *Enterococcus*, and *Acinetobacter* genera pose significant risks to humans; thus, their presence in animal samples cannot be ignored. The identified fungi, mostly opportunistic pathogens, pose a considerable risk primarily to immunosuppressed individuals [37].

The close cohabitation of many animals in shelters; the continuous intake of new, potentially infectious individuals; weakened immune systems; and the lack of treatment in stray animals all create and maintain favorable conditions for spreading *C. felis* infections [32]. An entirely separate quarantine area at the discussed cat shelter is not feasible, so the animals are isolated in cage quarantine. According to protocol, sick animals are separated in quarantine until recovery, and newly arriving animals are quarantined for a few weeks or months. This solution works relatively well but does not prevent the airborne transmission of other pathogens within the enclosed space, including feline herpesvirus, reovirus, calicivirus, etc. [38,39]. Unfortunately, the support for animal shelters in Hungary is quite limited today. They primarily rely on volunteers, donations, periodic tenders, or other alternative self-financing solutions to cover their expenses while performing vital work from public and animal health perspectives. In addition to rescuing and rehabilitating animals, they strive to control overpopulation [40]. However, being aware of the potential risks associated with shelters is crucial. The animals’ close and dense living conditions; the intake of new, potentially infectious individuals; and the challenges of providing individual quarantine can easily sustain infections within the population [32]. This poses a severe zoonotic risk not only to the animals but also to humans. Therefore, it is essential to develop the basic equipment, expansion, supplies, and hygienic conditions of shelters and to provide a more sustainable financial basis for their operation, primarily through state support. Making spaying more affordable or accessible would also be a significant milestone in addressing the problem [40].

Currently, there are no veterinary guidelines for the accurate monitoring of the treatment of chlamydial conjunctivitis using PCR. The PCR functioned exclusively as a supplementary confirmatory test, and the veterinarian did not always deem the confirmatory test necessary. In the case of chlamydiosis, studies or guidelines favor doxycycline therapy over, e.g., azithromycin in terms of efficacy. Moreover, they have found that 4 weeks should be sufficient for complete elimination of the organism. The continuation of treatment is recommended for two weeks after the resolution of clinical signs [41,42]. In our study, the veterinarian administered the treatment with a combination of rifampicin eye drops and doxycycline. It can be assumed that this may have contributed to the complete cure in 14 days with the resolution of clinical signs. Thus, the applied therapy proved successful for the animals participating in the study.

## 5. Conclusions

Based on this study, it can be concluded that the risk of chlamydiosis is present in animals. Due to their proximity to us, our pets can often be sources of infection, not only chlamydiosis but also other bacterial or fungal pathogens. From both human and veterinary health perspectives, it is essential to be aware of the potential for chlamydiosis and to thoroughly understand the circumstances and sources of zoonoses to address the issue effectively. Further studies involving parallel investigations in humans and animals would be necessary to better understand the risk of zoonoses.

## Figures and Tables

**Table 1:** Data of animals with positive pan-chlamydia PCR results with average Ct values, including the number of animals and their percentage distribution, sorted by relevant parameters.

Sources	Symptomatic Animals ^2^	Asymptomatic Animals ^2^	Total
Cat shelter (cats)	4 (4.3%)/8; Ct: 34.0	12 (12.9%)/35; Ct: 37.5	16 (17.2%)/43
Veterinary clinic (cats)	8 (8.6%)/20; Ct: 36.2	0 (0.0%)/0	8 (8.6%)/20
Veterinary clinic (dogs)	6 (6.5%)/13; Ct: 35.1	0 (0.0%)/0	6 (6.4%)/13
Household pets (cats)	1 (1.1%)/13; Ct: 38.1	1 (1.1%)/2; Ct: 38.8	2 (2.2%)/15
Household pets (dogs)	0 (0.0%)/2	0 (0.0%)/0	0 (0.0%)/2
Total ^1^	19 (20.4%)	13 (14.0%)	32 (34.4%)

^1^ Sum of the values in the cells corresponding to columns. ^2^ Number of positive samples/examined; average Ct values of positive animals.

## Data Availability

The data supporting this study’s findings are available from the corresponding author, Gabriella Terhes, upon reasonable request.

## Supplementary Materials

The following supporting information can be downloaded at: https://www.mdpi.com/article/10.3390/pathogens13090771/s1, File S1: Table of identified bacteria and fungi as a result of the culture-based examinations. File S2: Table of positive pan-chlamydia PCR results with relevant details. Figure S1: Melting analysis of pan-chlamydial PCR products using Gentier96E real-time PCR instrument (Xian Tianlong Science and Technology Co., Ltd., China). Samples 5-1 and 5-4 were *Chlamydia felis*; positive samples were confirmed by *C. felis*-specific PCR; positive control was *C. trachomatis* with Tm value 82.8.
