# Supplementary material for: The Occurrence of Chlamydia felis in Cats and Dogs in Hungary
Source: Pathogens. 2024 Sep 6;13(9):771. doi: 10.3390/pathogens13090771 (PMC11434856; doi:10.3390/pathogens13090771)

pathogens

MDPI

Article

The Occurrence of *Chlamydia felis* in Cats and Dogs in Hungary

Áron Balázs Ulbert [1], Hajnalka Juhász [1], Zsanett Karácsony [1], Katalin Bencze [2], Zoltán Deim [3], Katalin Burián [1] and Gabriella Terhes [1,*]

[1] Department of Medical Microbiology, Albert Szent-Györgyi Medical School, University of Szeged, 6725 Szeged, Hungary; ulbertaron@gmail.com (Á.B.U.); hajnus2007@gmail.com (H.J.); karacsony.zsanett@med.u-szeged.hu (Z.K.)
[2] Oxygen Animal and Environment Foundation, 6721 Szeged, Hungary; oxigen.asegitseg@gmail.com
[3] Dr. Zoltán Deim Veterinary Clinic, 6726 Szeged, Hungary; info@dmac.hu
* Correspondence: terhes.gabriella@med.u-szeged.hu; Tel.: +36-62545888

Supplementary File S1

Table of identified bacteria and funghi as a result of the culture-based examinations.

The distribution of identified species categorised by genera.

Bacteria			
**Genus**	**Species**	**Total (n)***	**%**
Achromobacter	*spanius* (1) **1-,1C,1H**	1	0.7
Acinetobacter	*pittii (1)* **1+,1C,1S**; *johnsonii (4)* **4-,4C,4S**; *schindleri (1)* **1+,1D,1V**; *radioresitens (1)* **1+,1C,1H**; *calcoaceticus (1)* **1-,1C,1H**; *ursingii (2)* **2+,1C,1D,2V**; *lwoffii (3)* **3-,3C,2S,1H**; *beijerinckii (1)* **1+,1D,1V**; *lactucae (1)* **1+,1C,1V**	15	9.8
Aerococcus	*viridans* (2) **2-,2C,2S**	2	1.3
Aeromonas	*caviae* (2) **1+,1-,2C,2S**; *veronii (1)* **1-,1C,1S**; *hydrophila (1)* **1-,1C,1S**	4	2.6
Agromyces	*bracchium* (1) **1-,1C,1H**	1	0.7
Bacillus	*cereus* (2) **2+,2C,1H,1S**; *pumilus* (2) **2+,2C,1V,1H**; *mycoides (1)* **1+,1D,1V**; *sonorensis (1)* **1+,1C,1H**	6	3.9
Brachybacterium	*conglomeratum (1)* **1-,1C,1S**	1	0.7
Brevundimonas	*diminuta* (3) **3+,1C,2D,3V**; *vesicularis (1)* **1+,1C,1V**; *sp. (1)* **1-,1C,1H**	5	3.3
Cellulomonas	*uda (1)* **1+,1D,1V**; *pakistanensis (1)* **1+,1D,1V**	2	1.3
Clostridium	*colicanis (1)* **1+,1C,1S**; *perfringens (1)* **1-,1C,1S**	2	1.3
Comamonas	*testosteroni (1)* **1+,1C,1S**	1	0.7
Corynebacterium	*mastitidis* (2) **1+,1-,2C,1S,1V**; *spheniscorum (1)* **1-,1C,1S**	3	2.0
Cutibacterium	*acnes* (1) **1-,1C,1S**	1	0.7
Enterobacter	*cloaceae* (4) **2+,2-,4C,3S,1H**	4	2.6
Enterococcus	*faecium* (3) **3+,3C,2S,1H**; *faecalis* (4) **3+,1-,4C,2S,2V**; *hirae* (1) **1+,1D,1V**; *italicus* (1) **1+,1C,1V**	9	5.9
Exiguobacterium	*mexicanum* (1) **1+,1D,1V**; *sp.* (2) **2+,2C,1V,1H**	3	2.0
Glutamicibacter	*arilaitensis* (3) **1+,2-,3C,2S,1V**; *protophormiae* (1) **1-,1C,1S**	4	2.6
Klebsiella	*pneumoniae* (3) **3+,2C,1D,2S,1V**; *aerogenes* (1) **1+,1D,1V**	4	2.6

Kurthia	*gibsonii (1)* **1-,1C,1S**	1	0.7
Lactococcus	*lactis (1)* **1+,1D,1V**; *raffinolactis (1)* **1-,1C,1S**	2	1.3
Lelliottia	*amnigena (1)* **1+,1C,1H**	1	0.7
Leuconostoc	*mesenteroides (1)* **1+,1C,1V**	1	0.7
Lysinibacillus	*fusiformis (1)* **1-,1C,1S**; *xylanilyticus (1)* **1+,1C,1V**	2	1.3
Massilia	*sp. (1)* **1-,1C,1S**	1	0.7
Microbacterium	*testaceum (1)* **1+,1D,1V**; *neimengense (1)* **1+,1C,1H**; *phyllosphaerae (1)* **1-,1C,1S**; *esteraromaticum (1)* **1-,1C,1S**; *oxydans (1)* **1+,1D,1V**; *paraoxydans (2)* **2+,2C,2V**; *foliorum (1)* **1+,1D,1V**; *liquefaciens (1)* **1+,1C,1V**; *luteus (1)* **1+,1C,1V**	10	6.5
Moraxella	*canis (1)* **1+,1D,1V**	1	0.7
Morganella	*morganii (1)* **1+,1D,1V**	1	0.7
Paenarthrobacter	*ilicis (1)* **1-,1C,1S**	1	0.7
Paenibacillus	*amylolyticus (2)* **1+,1-,1C,1D,1S,1V**	2	1.3
Pantoea	*agglomerans (5)* **5+,1C,4D,5V**	5	3.3
Proteus	*sp. (1)* **1+,1D,1V**	1	0.7
Pseudarthrobacter	*chlorophenolicus (2)* **2+,2C,2H**; *scleromae (1)* **1+,1C,1V**; *oxydans (1)* **1-,1C,1S**	4	2.6
Pseudescherichia	*vulneris (1)* **1+,1D,1V**	1	0.7
Pseudomonas	*aeruginosa (2)* **2+,2C,2V**; *koreensis (5)* **5+,3C,2D,4V,1H**; *corrugata (1)* **1+,1C,1H**; *taetolerans (1)* **1+,1C,1H**; *azotoformans (1)* **1+,1C,1H**; *extremorientalis (1)* **1+,1C,1H**; *agarici (1)* **1-,1C,1S**; *fulva (2)* **2+,1C,1D,1V,1H**; *rhodesiae (1)* **1+,1C,1H**; *chlororaphis (1)* **1+,1C,1H**; *flavescens (1)* **1-,1C,1S**	17	11.1
Psychrobacter	*pulmonis (2)* **2-,2C,2S**; *sanguinis (1)* **1+,1D,1V**	3	2.0
Rothia	*nasimurium (1)* **1+,1C,1V**	1	0.7
Serratia	*rubidaea (1)* **1-,1C,1S**	1	0.7
Sporosarcina	*ureae (1)* **1-,1C,1S**	1	0.7
Staphylococcus	*aureus (1)* **1+,1D,1V**; *felis (7)* **5+,2-,7C,3S,2V,2H,**; *epidermidis (3)* **2+,1-,3C,2S,1V**; *simulans (1)* **1+,1C,1V**; *sciuri (1)* **1+,1D,1V**; *haemolyticus (2)* **2-,2C,2S**; *pseudintermedius (1)* **1+,1D,1V**	16	10.5
Stenotrophomonas	*nitritireducens (1)* **1+,1C,1H**; *rhizophila (1)* **1+,1D,1V**	2	1.3
Streptococcus	*peroris (1)* **1+,1C,1V**; *sanguinis (1)* **1+,1C,1V**	2	1.3
Streptomyces	*naganishii (1)* **1+,1C,1V**	1	0.7
	Funghi		
Aspergillus	*flavus (1)* **1-,1C,1S**	1	0.7
Candida	*parapsilosis (1)* **1+,1C,1H**	1	0.7
Malassezia	*pachydermatis (2)* **2+,1C,1D,1S,1V**	2	1.3
Sarocladium	*strictum (1)* **1+,1D,1V**	1	0.7
Syncephalastrum	*racemosum (1)* **1+,1D,1V**	1	0.7
Trichophyton	*rubrum (1)* **1+,1C,1V**	1	0.7
**Total**		153	100.0

*number of species.

+, -, C, D, S, V, H indicate the main categories compared in the study. Microorganisms were characterised by these markers. **+**: symptomatic animal; **-**: asymptomatic animal; **C**: cats; **D**: dogs; **S**: cat shelter; **V**: veterinary clinic; **H**: household pets

Supplementary File S2

Table of positive pan-chlamydia PCR results with relevant details.

ID number	Ct values	Tm values	Evaluation
50586 +CV	40.00	84.2	positive
50907 +CS	34.80	84.8	positive
50908 +CS	37.60	81.3	positive
50909 +CS	28.50	81.6	positive*
50911 +CH	38.10	84.8	positive
51526 –CS	39.10	84.0	positive
51528 –CS	37.80	83.9	positive
51529 –CS	39.50	81.6	positive
53342 –CS	38.90	80. 0	positive
53340 -CS	39.70	82.9	positive
53339 –CS	38.30	81.6	positive
56423 +CV	35.40	85.1	positive
56411 +DV	38.90	83.0	positive
56410 +CV	39.20	85.2	positive
56413 +CV	35.50	84.6	positive
56918 +CV	39.90	84.8	positive
57394 +CV	37.50	82.5	positive
57895 -CH	38.80	83.1	positive
58538 +CV	21.20	82.1	positive*
151096 –CS	34.10	81. 9	positive
151102 +CS	35.00	84. 8	positive
151105 –CS	38.40	83. 7	positive
151108 -CS	38.20	82.0	positive
151336 +DV	40.00	81.0	positive
151338 -CV	39.50	81.4	positive
151339 +DV	39.50	81.3	positive
151671 +DV	38.50	82.8	positive
151673 +CV	37.70	82.0	positive
152373 +DV	28.90	81.7	positive*
152370 +DV	24.50	81.7	positive*
152868 -CS	35.60	81.3	positive
152875 -CS	35.20	82.2	positive
152876 -CS	34.60	81.8	positive

**C. felis* was detected by sequence analysis and confirmation by *C. felis* real-time PCR.

+, -, C, D, S, V, H indicate the main categories compared in the study.

+: symptomatic; -: asymptomatic; C: cat; D: dog; S: cat shelter; V: veterinary clinic; H: household pets; Tm: Melting temperature of pan-chlamydial PCR product.

Figure S1. Melting analysis of pan-chlamydial PCR products using Gentier96E real-time PCR instrument (Xian Tianlong Science and Technology Co., Ltd, China). Samples 5-1 and 5-4 were *Chlamydia felis* positive samples confirmed by *C. felis*-specific PCR; positive control was *C. trachomatis* with Tm value 82.8.

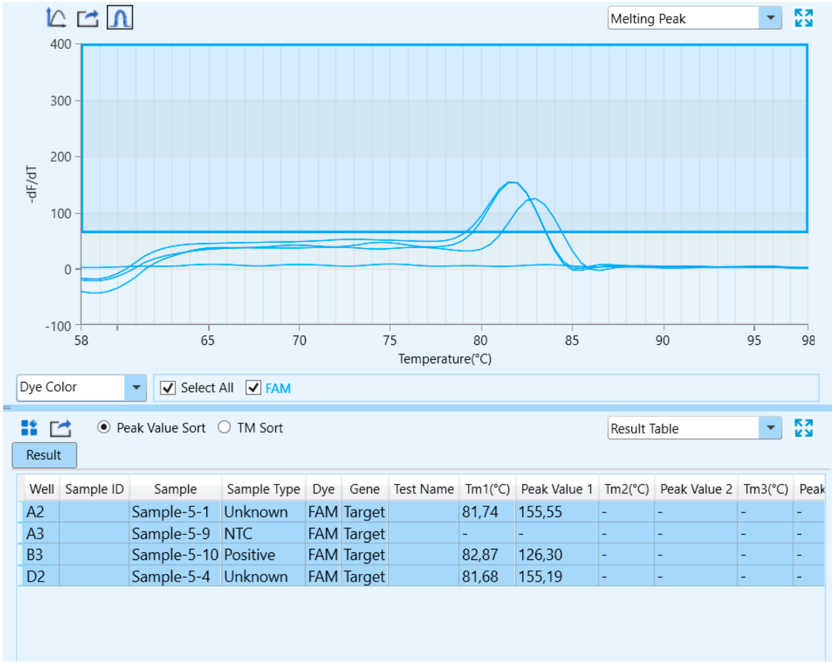

Supplement: Supplementary file 1 [file pathogens-13-00771-s001.zip › pathogens-3160703-supplementary.pdf]
